# COVID-19 outbreak: How do human resource management practices affect employee well-being?

**DOI:** 10.3389/fpsyg.2022.923994

**Published:** 2022-07-22

**Authors:** Elaina Rose Johar, Nadzirah Rosli, Siti Murni Mat Khairi, Shafiq Shahruddin, Norzanah Mat Nor

**Affiliations:** ^1^Faculty of Economics and Management, Universiti Kebangsaan Malaysia, Bangi, Malaysia; ^2^Faculty of Management, Education and Humanities, University College MAIWP International, Kuala Lumpur, Malaysia; ^3^Faculty of Business and Management, Universiti Teknologi MARA, Perlis, Malaysia; ^4^Arshad Ayub Graduate Business School, Universiti Teknologi MARA, Shah Alam, Selangor, Malaysia

**Keywords:** human resource management, employee well-being, AMO model, COVID-19, structural equation modelling

## Abstract

The study examines the effect of human resource practices (HRPs), including ability, motivation, and opportunity practices, on employee well-being (EWB) in the Malaysian environment during the COVID-19 pandemic. This research surveyed 154 service sector employees at Klang Valley, Malaysia. The data were analysed using structural equation modelling. Based on the ability, motivation and opportunity (AMO) theory, the results indicate that motivation and opportunity practices have a significant positive effect on EWB, whereas ability enhancing practices have an insignificant effect. Human resource policies and practices must foster a conducive yet contented work environment, and leaders must provide opportunities and motivation for employees to participate actively in the workplace. By doing so, the organisation’s value of human resources can be significantly increased, and the organisation’s goals can be achieved while employees’ overall well-being is enhanced, resulting in a win-win situation. This study uncovers the important roles of AMO practices that can effectively increase EWB.

## Introduction

The world witnessed an unprecedented event at the start of the year 2020. Nobody ever imagined that the entire world would enter a state of total lockdown, with people losing social, economic, and political ties and worse yet, no one was ever prepared for this to happen. It was a result of a sudden pandemic outbreak that people worldwide, particularly employees, were forced to adopt a new norm known as “working from home” (WFH), which was the most widely used term at the time. Fear of the COVID-19 virus, a lack of readiness to fully utilise technology, and adaptation to a new life norm have resulted in a slew of issues, most notably mental health issues. Numerous people have been laid off as a result of many businesses being forced to close due to the pandemic’s lockdown. Likewise, the COVID-19 pandemic has put a lot of attention into research calling especially the one that look into the effect on people’s behaviour (Islam et al., 2020; Ahmed et al., 2021; Malik et al., 2021). Therefore, the topic of employee well-being should be examined more closely during this challenging time.

The concept of well-being has been widely acknowledged as a national measure of productivity in both developed and developing countries (Ip, 2009; Caicedo et al., 2010; Miller, 2016). In keeping with Malaysia’s development objective for enhancing well-being outlined in the Eleventh Malaysia Plan 11MP (2016–2020), the government is dedicated to ensuring Malaysians’ sustained well-being through national social indicators. This is further emphasised in the Twelfth Malaysia Plan 12MP (2021–2025), which places a premium on social well-being as part of the Shared Prosperity Vision (SPV) 2030’s strategic thrust five (Loheswar and Jun, 2019). The SPV’s main objective is to ensure a decent standard of living for all Malaysians by 2030, making sure economic wealth and well-being for all Malaysians. Additionally, Malaysia, along with 192 other world leaders, adopted the 2030 Agenda for Sustainable Development (2030 Agenda) on 25 September 2015 at the United Nations General Assembly in New York. This is a worldwide commitment to more sustainable, resilient, and inclusive development, comprised of 17 Sustainable Development Goals (SDGs) and 169 targets. Malaysia recognises that achieving the SDGs comprehensively will require mobilising resources, such as manpower, capacity building, and physical space, as well as funding. Due to the fact that Malaysia’s national development plan has always prioritised economic, social, and environmental goals, the SDGs are being implemented in accordance with the 12MP. Five clusters are involved: inclusivity, well-being, human capital, environment and natural resources, and economic growth. The cluster of well-being includes Goal 3 of the SDGs, which is improved health and well-being (Economic Planning Unit Prime Minister’s Department Malaysia, 2021). When the COVID-19 pandemic spread through country, many people may not have noticed that employees’ health and well-being were given as much attention as they had been in the past.

The health and well-being of an employee, whether physical, mental or emotional, has a significant impact on the quality and success of a company (The Edge Market, 2020). The latest findings from the AIA Vitality 2019 Malaysia’s Healthiest Workplace study found a growing culture of overworking within Malaysian organisations could lead to the issues such as mental wellness, clinical health, work environment, and sleep. The survey findings also revealed that mental health issues are on the increase, with 22% of employees indicating that they are now dealing with a lot of financial difficulties. Furthermore, 20% of employees are still subjected to workplace bullying, which contributes to their stress at work (AIA Bhd, 2021). Furthermore, as according to Employment Hero’s (2021) report, “The Impact of COVID-19 on Mental Health in the Workplace”, which was focused on an analysis of over 1,000 Malaysian employers and employees. It was reported that 71% of Malaysian employees are concerned about their financial well-being, 66% about their physical health, and 62% about their psychological disorders. Moreover, according to the same report, the mental health concerns among Malaysian employees are (62%) as compared to other employees in countries such as Singaporeans (50%), the United Kingdom (49%), New Zealand (46%) and Australians (46%). About 70% of Malaysian employees hope they can have a healthy work-life balance. The survey also indicated the significance of well-managed communications and transitions to WFH. Prior to the pandemic, 59% of individuals who had been working remotely claimed being stressed. As a result of the pandemic, many have been obliged to WFH, where 64% reported being frustrated with the arrangement (Employment Hero, 2021). According to numerous reports, emotional, financial and physical health, or simply the well-being of employees, is still the most common concern encountered by employees in general. As workers transition into the new normal of working remotely, adequate human resource practices (HRPs) should be in place to address employee well-being problems.

Following the suggestion by Guest (2017) and the urgency to develop more study on employee well-being, this study had contributed to the literature by providing the resonate behind the implementation of AMO enhancing practices that help to strengthen the well-being of employee particularly in the pandemic situation. This study also verified that the right application of human resource practices is important and must be aligned with the current needs and wants of the employees. The practices that might useful before the pandemic started might not be well accepted in the present situation. As such, training practices in this study revealed that it was not applicable due to the new working norm of WFH and there was lack of training available or if there was training available online, employees were not in favour of it. Moreover, the majority of empirical studies to date have examined employee well-being as a mediator (Khoreva and Wechtler, 2018; Sivapragasam and Raya, 2018; Salas-Vallina et al., 2020, 2021) and have used multiple dimensions of well-being as the dependent variable (Zhang et al., 2020; Guerci et al., 2022) rather than unidimensional.

Hence, the purpose of this study is to determine the extent to which AMO practices impact the well-being of Malaysian employees, particularly during the COVID-19 pandemic. A study was done among 154 employees in Klang Valley, Malaysia. The exact research question to be answered in order to fulfil this research aim is: RQ1: Do AMO practices have an impact on employee well-being? The following sections outline the structure of this paper. It begins by emphasizing the organisation’s importance of employee well-being. It then discusses the probable utility of AMO practices in enhancing employee well-being, implying the importance of conducting empirical research on the relationship between AMO practices and employee well-being. Following that, hypotheses regarding the relationship between AMO practices and employee well-being are developed. This is followed by an explanation of the methodology and results. Following that, the study findings are examined, as are the contributions to the literature on employee well-being and AMO practices, as well as additional contributions. Finally, the limitations of the study, research implications, and practice implications are discussed.

## Literature review

### Employee well-being

In the literature, there are conflicting notions and definitions of employee well-being. Scholars use the phrase “well-being” equally with other ideas or terms such as “satisfaction”, “pleasure”, and “quality of life” (Kianto et al., 2016; Achour et al., 2017). Employee well-being is also referred to as workplace well-being or quality of working life (Chan and Wyatt, 2007). The term for what constitutes organisational well-being has evolved and widened over time. Miller (2016) defines well-being succinctly as “the point of equilibrium between an individual’s resource pool and the obstacles confronted”. Well-being has been extensively discussed by psychologists’ researchers, and psychologists have divided well-being into two distinct but related forms, such as hedonism and eudemonism (Chumg et al., 2015; Lomas et al., 2017). Hedonism, also known as subjective well-being, is concerned with personal well-being, which includes a desire to be accomplished, a good way to avoid pain, and a desire to be happy. Eudemonism-focused psychologists believe that an individual’s well-being is more contextual, nuanced, and important in life. Unlike hedonism, eudemonism is measured by psychological well-being (Chumg et al., 2016). Salanova et al. (2014) emphasise that psychological well-being refers to individuals’ assessments of their lives, both affective and cognitive. According to (Kooij et al., 2013), psychological well-being “is concerned with an individual’s subjective experience”. Additionally, Lomas et al. (2017) suggest that an individual’s well-being should encompass both subjective and psychological well-being, sometimes referred to as quality of life.

Employee well-being is emphasised, which may be defined as “individual life fulfilment and enjoyment” is the key to improving organisational performance (Huang et al., 2016). Employee well-being is characterised as a positive assessment of one’s life satisfaction and happiness, as defined by Chumg et al. (2015), Hills and Argyle (2002), and Mellor et al. (2016), who believe that well-being is concerned with people’s point of view on their life experiences, individual satisfaction, and emotional characteristics. In Malaysia, a study on manufacturing employees conducted by Wahab et al. (2021) discovered that well-being and work-life balance are not a major concern, particularly among young and low-skilled manufacturing workers who are only concerned with making more money and are ready to work long hours for overtime allowances. Individually, a study performed in a Malaysian higher education institution during the COVID-19 pandemic by Daud et al. (2020) discovered that a combination of emotional wellness, family interaction, organisational psychosocial support, and WFH affects employee well-being. Nevertheless, only mental health shows a non-significant link, possibly because employees are accustomed to working under difficult situations and possess the ability to multitask.

Additionally, employee well-being, according to Lin et al. (2014) has an impact on employees’ job performance. Employee work performance may be increased when people’s well-being is developed. Individuals with a low sense of well-being, on the other hand, will lack job focus, resulting in poor work performance. It has been demonstrated that there is a favourable correlation between employee well-being and organisational performance (Taris and Schreurs, 2009; Van den Bosch and Taris, 2014). Van den Bosch and Taris (2014) suggested that a higher degree of well-being among employees results in an increase in individual performance, which in turn results in improved organisational performance. Poor employee well-being can reveal negative attitudes and behaviours, such as decreased work performance, non-appearance, extended sick leave, and disloyalty to the organisation (Lin et al., 2014).

Besides that, employee well-being is also a critical component of individual and organisational success, and it is viewed as a significant result by policymakers and HR practitioners alike (Lin et al., 2014; Chumg et al., 2015; Kianto et al., 2016; Guerci et al., 2022). Cañibano (2013) examines the effects of applying an innovative, three-dimensional human resource management (HRM) strategy on employee well-being, including physical, psychological and social well-being. A qualitative study was undertaken, and the results revealed that innovative HRM practices can result in both positive and negative well-being outcomes. Aside from that, Huang et al. (2016) recognised high-performance work systems as one of the main management practices for promoting employee well-being.

### Human resources management practices

Historically, HRM has defined practises as a collection of practices instead of the more common process of strategically integrating human resource decisions (Duberley and Walley, 1995). Noe et al. (2010) define HRM as a philosophy, policy, system, and practices that are concerned with an employee’s behaviour, attitude, and performance. Indeed, certain practices serve as the foundation for the multiple practices proposed by numerous prior HRM scholars, as there is no consensus on what constitutes an HRM practice.

### Ability, motivation and opportunity (AMO) enhancing practices

Earlier research has looked at the “black box” of HRM’s link to performance, and one well-known factor is the AMO framework, which consists of three components that improve employee performance: individual ability (A), motivation (M) and opportunity to participate (O) (Appelbaum et al., 2000). In accordance with some authors, this model incorporates the basic psychological concept of (1) motivation as the driving force for behaviour; (2) ability as the skills and capabilities needed for behavioural performance; and (3) opportunity as a context and situational limitation relevant to behavioural performance (Maclnnis and Jaworski, 1989; Hughes, 2007; Kroon et al., 2013).

Accordingly, Boxall and Purcell (2011) observe that once they are able to accomplish the task (A), they are doing their job (M), and their workplace offers the essential support and outlets for expression (O). As a consequence, ability can be defined as a person’s ability to assist them in doing specific activities (Kim et al., 2015). Employee recruiting, formal training, and performance assessment are all examples of practices (Raidén et al., 2006; Kroon et al., 2013; Marin-garcia and Tomas, 2016). Training and development procedures assist in increasing the likelihood of acquiring new abilities, comprehending the problem, and identifying new viewpoints. Moreover, ability practices also assist in recruiting and selecting individuals who suit the profile of the organisation (Bos-Nehles et al., 2013; Schimansky, 2014; Marin-garcia and Tomas, 2016). On the other side, motivation practices refer to an employee’s desire to perform, which can be heightened by extrinsic or intrinsic motivation (Kim et al., 2015; Marin-garcia and Tomas, 2016). Motivation is frequently related with money or non-monetary incentives and the practices include incentives, career prospects, or performance evaluation (Raidén et al., 2006; Demortier et al., 2014; Munteanu, 2014). Accordingly, motivation practices if designed and implemented properly can make the employees feel valued by their organisation, and resulting in increased well-being. Furthermore, the opportunity is a collection of circumstances that take into account not just individual characteristics, but also the work environment that enables something to be accomplished (Marin-garcia and Tomas, 2016). As such, decision-making involvement, knowledge exchange, horizontal communication, and job development are all incorporated (Appelbaum et al., 2000; Schimansky, 2014).

### AMO practices and employee well-being

Zhang et al. (2020) demonstrated that three aspects of human resource management practises (HRMPs), namely (ability, motivation, and opportunity practices), had a beneficial effect on the three dimensions of EWB (life, job, psychological well-being). Additionally, their study merged hedonic and eudemonic perspectives and examined EWB in three dimensions: job, life, and psychological well-being. It was discovered that effective HRMPs not only benefit a certain type of employee well-being, but also can result in increased overall employee well-being.

Additionally, Salas-Vallina et al. (2021) discovered a relationship between AMO practices and well-being, where well-being was used as a mediator between AMO practices and organisational citizenship behaviour (OCB). Employee well-being acts as a moderator between AMO HRM practices and the OCB relationship. AMO HRM methods improve engagement and trust and reduce tiredness, indicating that they play a critical role in boosting OCBs.

The most recent study Guerci et al. (2022) compares AMO-enhanced high performance work practices (HPWPs) to three categories of employee well-being: health, happiness, and relational well-being. According to them, certain HPWPs initiate loss and gain cycles on distinct sorts of main resources, resulting in disparate correlations with relevant dimensions of employee well-being. Thus, HR practitioners concerned in enhancing employee well-being should direct investments toward activities that are genuinely related with employee well-being, and more specifically toward those dimensions of well-being that are of interest to the organisation.

## Hypotheses development and theory justification

Previous research has sought to explain the positive association between HRPs and EWB using behavioural theory (Peccei, 2004) and social exchange theory (Van de Voorde et al., 2012). This study took on a different strategy in this study, one that is based on the AMO theory. As per AMO theory, HR practices can be classified into three groups: ability-enhancing, motivation-enhancing, and opportunity-enhancing practices (Appelbaum et al., 2000). AMO theory contains three systems that outline individual characteristics in confirming that employees have the right skills, motivating employees to develop discretionary behaviours and empowering them towards organisational outcomes (Harney and Jordan, 2008; Abubakar Tabiu et al., 2016). The AMO framework in particular provides a comprehensive description of how HR practices can influence corporate performance through employees determination (e.g., formal staffing and training), motivation (e.g., formal performance evaluation and appropriate reimbursement), and participation opportunity (e.g., use of attitude investigations; Obeidat et al., 2016).

As such, this study was based on the relationship between HRPs, specifically AMO-enhancing practices and EWB. These practices were selected in light of two points made by Guerci et al. (2022): first, a significant drawback of the strategic HRM literature is that there is still no agreement on which HRM practices should be classified as HPWPs or under the ability-motivation-opportunity (AMO) framework, which is progressively used to categorise HPWPs (Lepak et al., 2006). According to this paradigm, each employee’s performance is determined by his or her abilities, motivation, and opportunity to perform (Appelbaum et al., 2000). Thus, companies should use HRM methods to guarantee that employees possess the essential skills, are highly motivated, and have numerous possibilities for engagement. Second, there is a continuing discussion in contemporary HRM literature over the source of data to be used for HPWPs. A recent review of the literature on that subject revealed that over the last two decades, HRM studies have increasingly relied on employees (rather than managers) as respondents (Beijer et al., 2019). Employee (rather than manager) data are deemed more appropriate for researching the impacts of HPWPs on employee attitudes and behaviours, as employees’ perceptions of HPWPs are temporary closer to, and thus more predictive of, their attitude and behaviour results (Kehoe and Wright, 2013). Additionally, the utilisation of employee data (rather than manager data) is consistent with the ethical imperative to place people at the centre of HRM research (Guest, 1999). As a result of these considerations, the hypotheses were created by focusing on AMO procedures as viewed by the individual employee and doing the empirical study utilising employee data. Therefore, this current study intends to fulfil the highlighted gap in the literature by proposing four hypotheses.

### Ability-enhancing practices and EWB

Ability-enhancing practices are aimed at enhancing employees’ knowledge and abilities to perform their jobs as expected, thereby contributing to the organisation’s success (Tharenou et al., 2007). These practices include hiring, training, and professional development. Ability practices may provide employees with necessary resources to achieve critical career outcomes for EWB. Hence, it is hypothesised that:

*H1*: Ability-enhancing practices have a positive effect on employees’ well-being.

### Motivation-enhancing practices and EWB

Motivation-enhancing practices are intended to increase employees’ extrinsic or intrinsic motivation to perform to expectations (Jiang et al., 2012). Extrinsic motivation refers to the external benefits an employee associates with engaging in their job while intrinsic motivation is the feeling of internal satisfaction and enjoyment of a person when engaging in their job. These include performance management procedures, compensation, bonuses, and incentives policies, and so on. If designed and implemented properly, employees will feel valued by their organisation, resulting in increased well-being. Thus, it is hypothesised that:

*H2*: Extrinsic motivation-enhancing HRMPs have a positive effect on employees’ well-being.

*H3*: Intrinsic motivation-enhancing HRMPs have a positive effect on employees’ well-being.

### Opportunity-enhancing practices and EWB

The term “opportunity practices” refers to those that enable employees to communicate their ideas, take ownership of creating goals, and completing assigned duties (Mathieu et al., 2006). These practices include employee participation and involvement in critical decision-making processes such as job design and goal planning, as well as decentralisation and increasing job autonomy (Jiang et al., 2012). By applying these HR enhancing practices, employees may be able to meet their self-achievement needs, resulting in increased EWB. Therefore, it is hypothesised that:

*H4*: Opportunity-enhancing HRMPs have a positive effect on employees’ well-being

**Figure 1:**
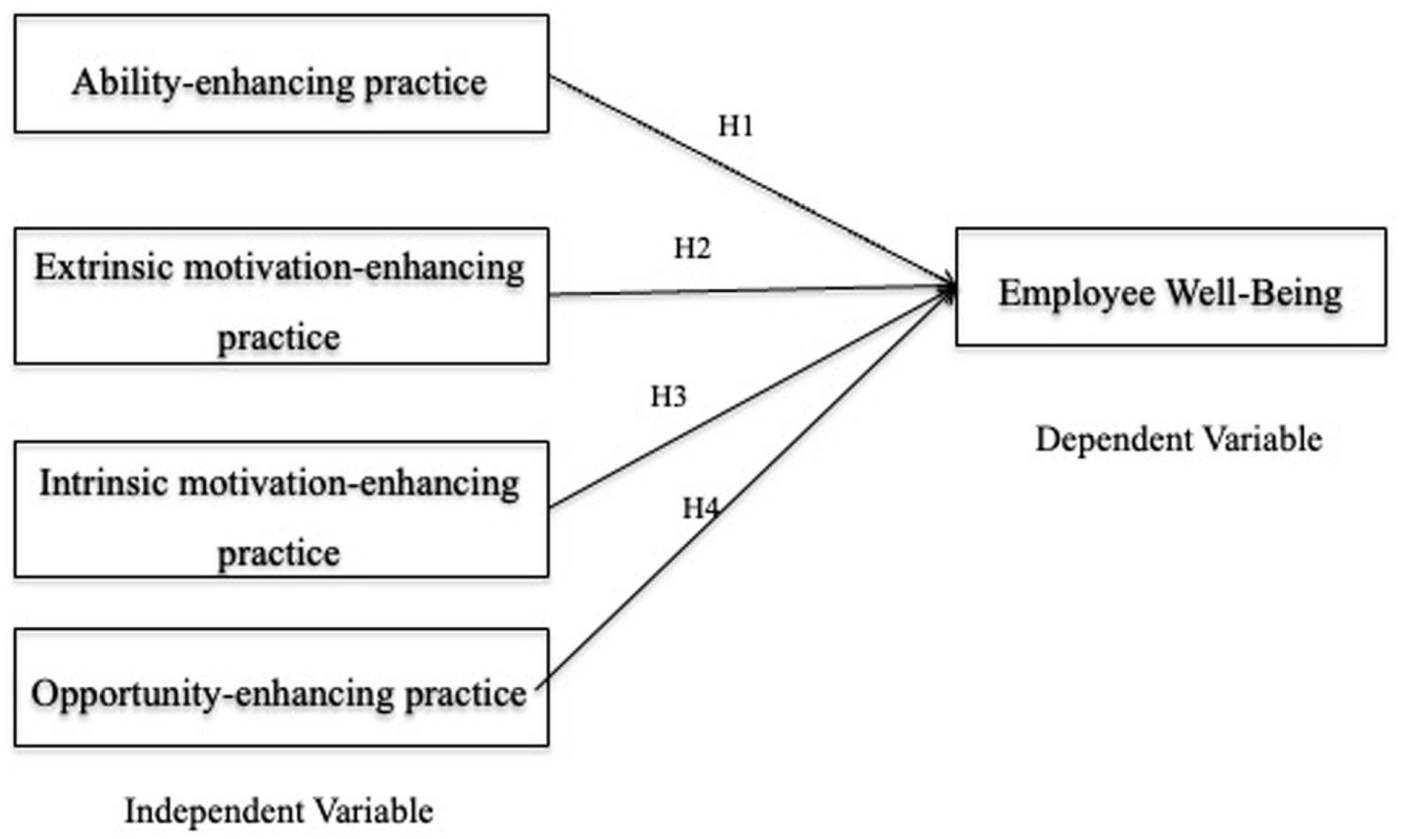
Theoretical framework.

In general, the relationship indicated above can be seen in Figure 1, our theoretical framework.

## Methodology

### Research setting and participants

The data were obtained in the second quarter of 2021 *via* an online survey of employees in the service sector in Klang Valley, Malaysia. Malaysia’s services sector accounts for more than half of the country’s GDP, making it a significant contributor to economic growth, efficiency, and earnings. The services market is open and well-regulated, facilitating access to information, expertise, technology, and financing while also facilitating the cross-border mobility of skilled labour (Malaysian Investment Development Authority, 2021). Accordingly, 8.352 million jobs were generated in the second quarter of 2021 across all economic sectors, with the service industry alone creating 4.312 million positions and 4.285 million paid employees. Klang Valley, Malaysia was selected as the population for this study because it had labour force participation rates above the national average of 74.9% (Department of Statistics Malaysia, 2021). Employees of various levels of classification were used as respondents in this study, and they are still actively employed at the moment. As a result, the unit of analysis for this study was the individual employee. Similarly, because there was no sampling frame of all service employees in the population, the convenience sampling approach was adopted in this study. The online survey questionnaires were distributed to the intended respondents *via* the social media platform. Respondents were also asked to forward the survey to other contacts in the various organisations. The G*Power sampling size determinant was used in this survey to determine sample size using research predictors (variables). This study’s model had four predictors. The minimum sample size required was only 85 when using G*Power with an effect size of 0.15, alpha of 0.05, and power of 0.8. Therefore, a total number of 300 questionnaires were disseminated, in which was more than the minimum sample size required and also to deal with the issue of non-response of the respondents. As a result, the study managed to get 52% of response rate. Hence, the sample size of 154 is deemed sufficient and exceeds the minimum sample size requirement.

The demographic analysis confirmed that the majority of respondents (64.3%) were females between the ages of 26 and 35 (51.3%). More than half of the respondents (56.5%) were married, 72.0% held executive or higher-level positions, and worked from home during the movement control order (MCO) (88.3%).

### Measures

#### Ability, motivation and opportunity enhancing HR practices

The AMO practices scale was developed from Andreeva and Sergeeva (2016) study on knowledge sharing among school teachers and adjusted for this study’s context. Sample items were: “my job specifically rewards my skills with monetary incentives” (extrinsic motivation enhancing HR practices); “to what extent is your job characterised by the following: the freedom to carry out my job the way I want to” (intrinsic motivation enhancing HR practices); “in our company there are trainings to develop interpersonal communication skills” (ability enhancing HR practices); and “the company invites high-performance employees to share their knowledge with others in meetings” (opportunity enhancing HR practices). The scale had 13 items that were scored on a 7-point Likert scale with answers ranged from 1 (“strongly disagree”) to 7 (“strongly agree”).

#### Employee well-being

The employee well-being scale from the Oxford Happiness Questionnaire (OHQ) formed by Hills and Argyle (2002) was used in this study as suggested by Chumg et al. (2016). The OHQ was originated from the Oxford Happiness Inventory (Argyle et al., 1989) which has received widespread praise for its strong construct validity and reliability (Hills and Argyle, 2002; Robbins et al., 2010). This scale had eight questions on it. The respondents were asked to rate their level of agreement on a 7-point Likert scale ranging from 1 (“strongly disagree”) to 7 (“strongly agree”).

Table 1 displays the statements used for all the research questionnaire’s selected items. As previously stated, the literature validated all the selected items.

**Table 1:** Constructs/items used in the research’s questionnaire.

Construct	Definition	Item	Adapted from
Employee well-being	The degree to which an employee feels a sense of well-being in the organisation (Oxford Happiness Questionnaire Short-Form).	WB1: I do not feel particularly pleased with the way I am. WB2: I feel that life is very rewarding. WB3: I am well satisfied about everything in my life. WB4: I do not think I look attractive WB5: I find beauty in some things. WB6: I can fit in everything I want to. WB7: I feel fully mentally alert. WB8: I do not have particularly happy memories of the past.	Hills and Argyle, 2002
Extrinsic motivation	The external benefits an employee associates with engaging in their job.	EM1: My job specifically rewards my skills with monetary incentives. EM2: My job specifically rewards my skills with non-monetary incentives. EM3: In my office, employee’s skills are a component in employees’ performance evaluation.	Andreeva and Sergeeva, 2016
Intrinsic motivation	The feeling of internal satisfaction and enjoyment of a person when engaging in their job.	IM1: The freedom to carry out my job the way I want to. IM2: The opportunity for independent initiative in performing my job. IM3: High level of variety in my job.	Andreeva and Sergeeva, 2016
Ability	Certain skills employees possess in order to be able to effectively explicate and transfer what they know.	AB1: In our company there are trainings to develop interpersonal communication skills. AB2: In our company there are trainings for teamwork skills. AB3: The company provides trainings to develop skills of self-reflection and knowledge externalization.	Andreeva and Sergeeva, 2016
Opportunity	Organizations provide employees with appropriate opportunities to apply their skills and motivation	OPP1: The company holds birthday parties, trips, and other hours together activities that promote friendship among colleagues. OPP2: The company invites high-performance employees to share their knowledge with others in meetings. OPP3: The company invites employees who have just acquired new knowledge from outside sources to share what they have learned. OPP4: The company holds regular meetings where colleagues can share successful experiences to resolve work problems.	Andreeva and Sergeeva, 2016

## Data analysis and results

Due to the survey nature of the data, multivariate normality was determined using the web-based software^1^ as suggested by Cain et al. (2017). The Mardia coefficient of multivariate skewness was 4.401 and the kurtosis was 41.608 (with cut-off values of ± 1 and ± 20, respectively, DeCarlo, 1997), indicating that the data were not multivariate normal. As a result, SmartPLS 3.3.7, a second-generation structural equation modelling (SEM) software, was chosen to perform bootstrapping on the model. Over the last 20 years, many researchers have increasingly turned to second-generation techniques and this method is referred to as SEM, which enables researchers to incorporate unobservable variables measured indirectly by indicator variables (Hair et al., 2017). In addition, SEM also facilitates the accounting of the error of measurement in the observed variables (Chin, 1998). The measurement model was evaluated first, followed by the structural model, as suggested by Ramayah et al. (2018) and Hair et al. (2019).

Due to the fact that data were gathered from a single source, a full collinearity analysis was conducted to determine whether common method bias was a concern in our study, as suggested by Kock and Lynn (2012). Firstly, a dummy variable was established in Excel using the random function; subsequently, all of the constructs (such as the dependent variable) were regressed in the research model against this common variable. The results in Table 2 indicate that there was no cause for serious concern, as all VIFs were less than the 3.3 threshold.

**Table 2:** Full collinearity estimates.

	Ability	Employee well-being	Extrinsic motivation	Intrinsic motivation	Opportunity
VIF	1.908	1.739	1.450	1.805	2.184

Additionally, we used the marker variable technique to address the issue of method variance. This study included three elements from workplace family-supportive programmes by Frone and Yardley (1996) that were gathered in the same survey but not included in the model under examination: (1) “After work, I come home too tired to do some of the things I’d like to do”, (2) “My job takes up time that I’d like to spend with family/friends”, and (3) “My job interferes with my responsibilities at home, such as cooking, cleaning, shopping etc”. These were used as indicator markers. This study utilised the partial correlation method with a theoretically unrelated marker variable, as recommended by Podsakoff et al. (2003), in order to examine the study’s common method bias. Despite the fact that Podsakoff et al. (2003) provided two methods to partial correlation methods: partialling out of social desirability items and partialling out of unrelated marker variables. Since unrelated marker variables can also include social desirability items, both approaches are similar. Partialling a “marker” variable is analogous to partialling a general factor. The only distinction is that the partial extraction of latent marker variables occurs in place of the general factor. The study used Smart PLS software to create a hypothesised model and then measured the *R*^2^ value of an endogenous construct. The study then partially removed the marker variable from the endogenous construct and calculated its *R*^2^ value again. The difference between the *R*^2^ values of the endogenous construct before and after the marker variable was then compared (e.g., 0.476–0.425 = 0.051). Thus, the difference in the *R*^2^ value of the endogenous construct after partial exclusion of the marker variable was 0.051, which is not statistically significant. This finding adds to the evidence that there was no significant common method bias in this study.

### Measurement model

To ensure that the measurement items were valid and reliable, the loadings from the results, as well as the average variance extracted and composite reliability, were evaluated. In order to meet the threshold of all the assessments involved, all the criteria for measurement model had to be established in this study. The evaluations begin by examining the reliability of internal consistency. After establishing the reliability of the construct, the research study had to assess the convergent validity. Lastly, in order to evaluate the structural model, the establishment of discriminant validity had to be succeeded.

The first criterion assessed in this study was the reliability of internal consistency. Composite reliability takes into account the different outer loadings of the indicator variables. Composite reliability varies from 0 to 1 and the higher values describe higher levels of reliability in the research study. The threshold for composite reliability is 0.7. Composite reliability values below 0.6 indicate a lack of internal consistency in reliability (Drolet and Morrison, 2001; Rossiter, 2002; Hayduk and Littvay, 2012).

For the next stage of the assessment of the measurement model, the research had to analyse the convergent validity of all indicators in the construct. As stated in Hair et al. (2017), the convergent validity is the extent to which the measure is positively correlated with the alternative measure of the same constructs. Convergent validity had to be assessed for the outer loading of the indicators and the average variance extracted (AVE). The outer loading size is also known as the reliability of the indicator. The thumb rule for reliability of the indicator should be 0.7 or higher. The square of the standard indicator’s outer loading represents how much of the variation in an items is explained by the construct, and this is also described as the variance extracted from the item. Indicators below the external load value of 0.7 should be eliminated; however, the effect of their removal should be carefully examined. Indicators with an outer loading of 0.4–0.7 should be considered for removal from the scale, only when it is lead to higher AVE according to the threshold indicated above. However, the indicators with an outer loading below 0.4 are always removed from the construct (Bagozzi et al., 1991; Hair et al., 2011). As for AVE, this criterion is defined as the grand mean value of the squared loading of the indicators associated with the constructs. The threshold for AVE is 0.5 or higher. The value means that the construct has explained more than half of the variance of its indicators. As shown in Table 3, all of the loadings were greater than 0.708, all of the AVEs were greater than 0.5, and all of the CRs were greater than 0.7, indicating that all of the measurements are valid and reliable (Ramayah et al., 2018; Hair et al., 2019).

**Table 3:** Measurement model.

Construct	Items	Loadings	CR	AVE
Employee well-being	WB2 WB3 WB5 WB6 WB7	0.867 0.861 0.441 0.639 0.777	0.846	0.535
Extrinsic motivation	EM1 EM2 EM3	0.724 0.744 0.843	0.814	0.594
Intrinsic motivation	IM1 IM2 IM3	0.832 0.821 0.756	0.843	0.642
Ability	AB1 AB2 AB3	0.893 0.932 0.919	0.938	0.836
Opportunity	OPP1 OPP2 OPP3 OPP4	0.785 0.887 0.893 0.895	0.92	0.743

After both the reliability of the indicators and the AVE met the threshold requirement, the research study then proceeds to the next assessment, which is discriminant validity. As explained by Hair et al. (2017), discriminant validity is the extent to which empirical standards make a construct truly different from other constructs. Establishing of discriminant validity indicated that the construct is unique and captures phenomena not represented by other constructs in the model. There have been a few approaches to the treatment of discriminant validity in the research study, such as cross-loading, the Fornell-Larcker criterion and the hetero-train-monotrait ratio (HTMT). As an overview of the first approach, cross-loading is basically an indicator’s outer loading on the associated construct and should be greater than any of its cross-loading on other constructs. Fornell-Larcker criterion is the second approach to the assessment of the discriminant validity of the model. Generally, the Fornell-Larcker criterion compares the square root of the AVE values to the latent variable correlations. The square root of each AVE construct should be greater than its highest correlation with any other construct. If this threshold is met in the model that has been examined, the discriminant validity has been established. Over the last few years, both approaches, cross-loading performance and the Fornell-Larcker criterion have been found untrustworthy in identifying discriminating issues of validity (Henseler et al., 2015). In order to address the related issue, Henseler et al. (2015) proposed the assessment of the heterotrait–monotrait ratio (HTMT) of the correlations. HTMT is the mean of all correlations of indicators across constructs that measure different construct relative to the mean of the average correlation of indicators that measure the same constructs.

For this research study, the HTMT approach was used to assess the validity of discriminant. The threshold value for HTMT is 0.9 if the path model includes constructs, which are conceptually very similar. The HTMT value above 0.9 indicates that the constructs lack discriminant validity. As Henseler et al. (2015) pointed out, a conservative threshold value of 0.85 appears to be warranted. In order to establish the discriminant validity, all the constructs involved in the research study had to comply with the threshold value requirement. In order to further evaluate the HTMT, the research study had to examine the HTMT ratio using the bootstrapping procedure to determine the distribution of the HTMT statistic. By doing so, the confidence interval for bootstrapping can be derived and the confidence interval containing the value 1 indicates that the constructs lack the discriminant validity. When the HTMT ratio inspection met the requirement for a confidence interval, the constructs were established and the structural model assessing assessment proceeded. Following that, the discriminant validity was determined using the HTMT criterion proposed by Henseler et al. (2015). If the ratios were less than HTMT_0.85_, it could be concluded that all measures were discriminant. Additionally, Franke and Sarstedt (2019) stated that if the upper limit of the HTMT bootstrapping value is not equal to 1, the measures are discriminant. As showed in Table 4, all ratios were less than 0.85; thus, the measures are distinct.

**Table 4:** Discriminant validity (HTMT ratios).

	1	2	3	4	5
1. Ability					
2. Employee well-being	0.554				
3. Extrinsic motivation	0.492	0.567			
4. Intrinsic motivation	0.724	0.688	0.568		
5. Opportunity	0.702	0.675	0.645	0.69	

### Structural model

The data analysis of this study continues with the analysis and focuses on the structural model that represent the underlying structural theory of the path model in the research study. Once the research study has confirmed and verified that all the constructs previously measured are reliable and valid, the next stage to be assessed is the results of the structural model. Essentially, the assessment of the structural model involved examining the model’s predictive capabilities and the relationships between the constructs in the path model. Hair et al. (2017), pointed out that, before describing the result of the structural model, there is a need for a few examinations, such as the assessment of collinearity, the assessment of the coefficient of determination and the size of the effect. Following the assessment of the collinearity, the coefficient of determination and the size of the effect of the constructs, the research study proceeded to determine the significance and relevance of the structural model relationship by analysing the results of the path coefficients based on the objectives and hypotheses of the research study.

Collinearity assessment is an evaluation of the correlation in the structural model between two constructs or predictive (Hair et al., 2017). For each subpart of the structural model, the research study had to examine each set of predictor constructs separately. It is very important to check the critical levels of collinearity between each set of predictive variables: ability, intrinsic motivation, extrinsic motivation and opportunity as predictors of employee well-being. A related measure of collinearity is the variance inflation factor (VIF), which was defined as the reciprocal of the tolerance (i.e., VIF = 1/TOL). TOL is represented as tolerance, the amount of variance of one formative indicator not explained by the other indicators. The threshold value for collinearity is VIF above 0.2 and below 5. In the context of PLS-SEM, a tolerance value below 0.2 and above 5 would indicate a potential collinearity problem (Hair et al., 2011). If the collinearity of the predictive construct did not meet the threshold value, consideration should be given to eliminating the construct, merging the predictive into a single construct, or creating higher-order constructs to treat the critical level of collinearity in the research study (Hair et al., 2017).

The next stage in the assessment of the structural model was to evaluate the coefficient of determination or, in other words, the R^2^ value. Hair et al. (2017) indicated that the determination coefficient is a measure of predictive power of the model and that it is the square correlation between the actual and predicted value of the particular endogenous construct. In addition, the coefficient also represents the combined effects of the exogenous latent variables on the endogenous latent variables. This can be explained by the fact that the coefficient is the amount of variance in the endogenous constructs explained by all the exogenous constructs associated with it (Hair et al., 2017). In this research, the endogenous constructs were the employee well-being, otherwise the independent variables (ability, intrinsic motivation, extrinsic motivation and opportunity) were the exogenous constructs. The reason for using *R*^2^ value is because it is the squared correlation between actual and forecast values and includes all of the data used for model estimation to assess the predictive power of the research model (Rigdon, 2012; Sarstedt et al., 2014). As indicated by Hair et al. (2017), *R*^2^ values range from 0 to 1, with higher values to 1 indicating a higher level of predictive power. The threshold for this assessment was 0.75, 0.50 and 0.25, respectively, which were explained as substantial, moderate and weak (Henseler et al., 2009; Hair et al., 2011).

The effect size is commonly and increasingly encouraged to be evaluated in the research study. As explained by Hair et al. (2017), the effect size (*f*^2^) is a change in the *R*^2^ value when the specified exogenous construct is omitted from the model. This can be used to assess whether the omitted construct has a substantive impact on the endogenous constructs (dependent variables). The thumb rule for evaluating the effect size, *f*^2^, is 0.02, 0.15 and 0.35, respectively, representing the small, medium and large effect of the latent endogenous variable (employee well-being; Cohen, 1988). The effect size, *f*^2^, values less than 0.02 can be described as having no effect on exogenous latent variable.

Thus, Table 5 illustrates that, the VIF values for all the predictive variables are clearly below the threshold of 5, the *R*^2^ values of employee well-being (0.4250) is considered as moderate and the effect size of ability showed no effect with the value of 0.003 on employee well-being, extrinsic motivation and intrinsic motivation showed small effect with the value of 0.028 and 0.073 on employee well-being, respectively, while opportunity also showed small effect on employee commitment with the value of 0.088.

**Table 5:** Coefficient of determination (*R*^2^), collinearity assessment (VIF) and effect size (*f*^2^).

	*R* ^2^	VIF EWB	*f* ^2^ EWB
1. EWB	0.425		
2. Ability		1.873	0.003
3. Extrinsic motivation		1.421	0.028
4. Intrinsic motivation		1.727	0.073
5. Opportunity		1.783	0.088

To estimate the structural model, a bootstrapping procedure with 5,000 resamples was run to generate the path coefficient, *t*-values, value of ps, and standard errors. Moreover, Hahn and Ang (2017) argued that value of ps are insufficient as a criterion for determining the significance of a hypothesis and recommended combining value of ps, confidence intervals, and effect sizes. Table 6 summarises the criteria used to test the developed hypotheses. First, this study looked at the factors that link to employee well-being which is ability, extrinsic motivation, intrinsic motivation and opportunity. The extrinsic motivation, intrinsic motivation and opportunity have a direct relationship with employee well-being (Table 6). This is because the t value is higher than the critical value, 1.645 at 5% significance level, the value of p of this relationship is lower than the significance level of 0.05, and the confidence interval for the relationship also shows a similar result, which does not include zero. Thus, the hypotheses H2, H3 and H4 are supported. On the other hand, ability show insignificant direct relationship towards employee well-being. Hence, the hypothesis H1 is not supported. To sum, ability (*R*^2^ = 0.425, *β* = 0.062, *p* = 0.271), extrinsic motivation (*R*^2^ = 0.425, *β* = 0.151, *p* = 0.028), intrinsic motivation (*R*^2^ = 0.425, *β* = 0.266, *p* = 0.002), and opportunity (*R*^2^ = 0.425, *β* = 0.318, *p* = 0.001) were all positively related to employee well-being. As a result, the findings indicate that HRM practices explained approximately 42.5% of the variance in employee well-being.

**Table 6:** Hypotheses testing.

Hypotheses	Relationship	Std beta	Std error	*t*-Value	*p*-Value	95% BCI LL	95% BCI UL	*f* ^2^
H1	Ability → EWB	0.062	0.101	0.61	0.271	−0.097	0.222	0.003
H2	Extrinsic motivation → EWB	0.151	0.079	1.917	0.028	0.021	0.27	0.028
H3	Intrinsic motivation → EWB	0.266	0.093	2.855	0.002	0.088	0.4	0.073
H4	Opportunity → EWB	0.318	0.103	3.096	0.001	0.15	0.483	0.088

### Model fit

To assess the overall model fit, this study used the bootstrap-based test for exact overall model fit. The results displayed in Table 7 show that the values of the discrepancy measures which is, geodesic distance (*d*G), SRMR, and squared Euclidean distance (*d*ULS), are below the corresponding critical value, namely the 95% quantile of the corresponding reference distribution. Hence, the results conclude that the specified model adequately fits the collected data. As such, the proposed model captures the available information in the data acceptably.

**Table 7:** Model fit.

	Saturated model	Estimated model
SRMR	0.05	0.05
d_G	0.265	0.265
d_ULS	0.304	0.304

## Discussion

The purpose of this study was to learn more about how HRPs namely the AMO practices affect EWB. A conceptual model with hypotheses was developed on the basis of the AMO theory. The findings suggest that employees who have experienced good HRPs will have greater well-being and satisfaction at the job. Employees need more motivation at job especially during hard time such as the current pandemic of COVID-19 to stay happy and well. Hence, strong support and motivation in terms of intrinsic or extrinsic motivation from employer is vital. The proper set of HRPs offered to employees will lead to higher performance and satisfaction. On the same note, the suitable opportunity enhancing practices should be in place in order to foster well-being of employees. Therefore, employer should be encouraged to promote meetings and get together activities or sessions with all the employees to enhance communication among them. When they are given opportunity to take part on such activities, they will feel happy and content. The results are aligned with the theoretical arguments of previous studies that AMO practices positively impact on EWB (Khoreva and Wechtler, 2018; Zhang et al., 2020; Guerci et al., 2022).

Nevertheless, ability practice has no effect on the relationship with EWB, in line with the study by Guerci et al. (2022), who discovered that training was not related to any of the EWB dimensions. The result suggests that in the current situation of COVID-19 pandemic, employer offers less training to their employees might due to the lack of readiness to face this unprecedented event. Fear of employees’ tardiness and unreadiness of employees to WFH could also be the reason of such insignificant result. Lack of teamwork to share knowledge and skills as before the pandemic happens lead to this finding. Unlike before the pandemic, many researches in the past concluded that ability enhancing practices specifically training will lead to higher performance and EWB.

Additionally, the findings of this study also show that, under the AMO model, it is preferable to regard the various dimensions of HR practices as three distinct components of an HRP system rather than as an interchangeable unidimensional frame for forecasting employee well-being (Zhang et al., 2020).

### Theoretical implications

This study makes two major theoretical contributions. First, we add to the literature by widening the evaluation of HRPs to include three dimensions: ability, motivation, and opportunity-enhancing practices, which were previously mostly used in HPWS research. Although the AMO framework was used in the vast majority of previous studies to understand the effect of HRPs on performance, empirical studies on the relationship between AMO dimensions of HRPs and EWB were scarce. Our findings indicate that the motivation and opportunity dimensions of HRPs have a significant impact on EWB, whereas the ability dimension has an insignificant impact. The findings support Pawar (2016) contention that HRMPs may be beneficial to employees’ well-being.

Second, our study contributes to the field of study by taking an integrative approach to EWB research. To be more precise, we combined hedonic and eudemonic perspectives and examined EWB as a dimension. By contrast, many previous studies have presented a fragmented picture of EWB by examining its dimensions separately, such as job satisfaction, physical well-being, and psychological well-being (Pawar, 2016; Khoreva and Wechtler, 2018). These studies fall short of providing a comprehensive picture of the antecedents of EWB. This current study affirmed the integrative perspective of EWB research by demonstrating that effective HRPs not only benefit a specific type of EWB, but also can result in increased overall employee well-being.

Thirdly, this study extends the People and Performance framework by Purcell et al. (2003), that used AMO framework as a mediating component between HRPs and HR related outcomes which includes organisational commitment, motivation and job satisfaction. By incorporating the AMO elements in the HRPs, this study looking into its relationship towards job satisfaction which is the employee well-being in this case. Thus, this study revealed that AMO enhancing practices can boost the well-being of the employees.

### Practical implications

This study’s findings have a number of practical implications. To begin, organisations must develop well-structured and meticulous HRM policies and ensure the implementation of effective practices. Organisational leaders can facilitate more effective and efficient human resource policies by viewing employees as assets rather than liabilities, investing in them, and focusing on their growth, survival and personal development. Meanwhile, leaders could provide opportunities and motivation for employees to actively participate in the workplace, increasing the value of human resources for the organisation, allowing the organisation to achieve its goals while also ensuring employees’ overall well-being, resulting in a win-win situation.

Second, organisations should reconsider the implementation of employee training, as the findings show no support for EWB, particularly when employees work from home. This could serve as a wake-up call to management, as they need to upgrade the training modules to meet the industry most recent and up-to-date skill requirements, especially in this industrial revolution and digitalisation era. Failure to keep up with the latest updates to employee knowledge and skill requirements will eventually result in employee dissatisfaction and low motivation to attend training. The training provided should also be appropriate for employees who work from home in order to avoid burnout and fatigue. Additionally, the insignificant result could be due to a lack of training provided during the pandemic and the need to take into account standard operating procedures (SOPs).

Thirdly, organisations must place a greater emphasis on motivational and opportunity-enhancing practices, as working conditions have evolved into the new norm. Working from home is a novel concept in some countries, and it was implemented for the first time in certain organisations. As a result, organisations should carefully structure their HRPs to align with the new norm, particularly in terms of employee motivation and opportunities to participate in organisational activities. Physical meetings and face-to-face interaction have now taken on a virtual form, which may result in a lack of personal communication and support among colleagues. This may not be apparent if employees have family at home, but what about employees who live alone and rely solely on workplace social interaction (Carnevale and Hatak, 2020). Finally, when organisations do not pay close attention to this issue, it is possible that it will affect the employees’ well-being.

## Conclusion

The study has added to the existing body of knowledge in order to achieve a higher level of employee well-being during Malaysia’s unimaginable pandemic. The importance of motivation and opportunity enhancement practices in helping employees improve their well-being was discovered. This type of assistance is critical in connecting HRPs to well-being and enhancing the quality of working life (Guest, 2017). Nevertheless, ability enhancing practices did not support employee well-being in this study, which could be a result of the new norm of working from home and also a lack of training provided during the pandemic due to the large number of SOPs to be followed. Additionally, rather than using unidimensional practices to measure employee well-being, AMO-enhancing practices can be used to represent multiple dimensions of HRPs. Previously, a substantial amount of research has examined the AMO model in conjunction with HPWPs and its relationship to organisational performance. Thus, this study established that AMO-enhancing practices have a significant impact on employee well-being, even in light of the current global economic downturn.

## Limitation and future directions

Although the findings revealed two strong relationships for achieving employee well-being, this study has several limitations. First, the study had a small sample size, though it was statistically significant. A larger sample size may be considered in future studies to improve generalisability. Second, the study was cross-sectional; future studies should include longitudinal settings or use current data collection methods, such as daily diary method. Furthermore, future studies could test the current model in other industries and countries, as well as conduct cross-country comparisons, to improve the generalisability of the results as the world faces the same unprecedented pandemic situation. Furthermore, future studies are suggested to advance the literature of the AMO model and employee well-being. Future studies may expand on this research by looking into the mediating or moderating effect of the relationships (Nielsen et al., 2017) as well examining other relative impact of AMO such as commitment and retention of employees.

## Data availability statement

The original contributions presented in the study are included in the article/supplementary material, further inquiries can be directed to the corresponding author.

## Author contributions

EJ and SK designed the research. SK and NN collected the data. EJ conducted the data analysis. NR and SS wrote the manuscript. All authors contributed to the article and approved the submitted version.

## Conflict of interest

The authors declare that the research was conducted in the absence of any commercial or financial relationships that could be construed as a potential conflict of interest.

## Publisher’s note

All claims expressed in this article are solely those of the authors and do not necessarily represent those of their affiliated organizations, or those of the publisher, the editors and the reviewers. Any product that may be evaluated in this article, or claim that may be made by its manufacturer, is not guaranteed or endorsed by the publisher.

## Footnotes

^1^ https://webpower.psychstat.org/models/kurtosis/
